# Bibliometric analysis of the association between drinking water pollution and bladder cancer

**DOI:** 10.3389/fonc.2023.1170700

**Published:** 2023-06-29

**Authors:** Ying Zhang, Mei Liu, Jiajun Wang, Kexin Han, Fuyu Han, Bicheng Wang, Si Xie, Chunhui Yuan, Mingdeng Zhao, Shuo Li, Jun Wang

**Affiliations:** ^1^ State Key Laboratory of Water Resources and Hydropower Engineering Science, Wuhan University, Wuhan, China; ^2^ Center for Single-Cell Omics and Tumor Liquid Biopsy, Zhongnan Hospital of Wuhan University, Wuhan, China; ^3^ Department of Laboratory Medicine, Wuhan Hankou Hospital, Wuhan, China; ^4^ Department of Laboratory Medicine, Zhongnan Hospital of Wuhan University, Wuhan, China; ^5^ Department of Pathology, Zhongnan Hospital of Wuhan University, Wuhan, China; ^6^ Department of Laboratory Medicine, Wuhan Children’s Hospital (Wuhan Maternal and Child Healthcare Hospital), Tongji Medical College, Huazhong University of Science and Technology, Wuhan, China; ^7^ Department of Clinical Laboratory, Renmin Hospital of Wuhan University, Wuhan, China

**Keywords:** drinking water pollution, bladder cancer, bibliometrics analysis, co-occurrence, future direction

## Abstract

**Background:**

Bladder cancer has become an increasingly intractable health problem worldwide. Long-term drinking water pollution is known to promote its occurrence. This study aimed to analyze the research status, hot spots, and future trends of drinking water pollution and bladder cancer through extensive bibliometric examination to provide reference data for better prevention and management of bladder cancer.

**Methods:**

The Scopus database developed by Elsevier was browsed for articles that met the predefined criteria using the search terms related to drinking water and bladder cancer. Included articles were further evaluated by year of publication, subject category, institution, article type, source journal, authors, co-authorship networks, and text mining of titles by R software packages tm, ggplot2 and VOSviewer software.

**Results:**

In total, 687 articles were selected after a comprehensive literature search by the Scopus database, including 491 research articles, 98 review articles, 26 conference papers, 23 letters and 49 other documents. The total number of articles published showed an upward trend. The United States has the largest number of published articles (345 articles), institutions (7/10) and funding sponsors (top 5). The journal with the most publications was *Environmental Health Perspectives*, with 46 published. The highest number of citations up to 2330 times for a single article published in 2007 on the journal of *Mutation Research*. Professor Cantor K.P. was the highest number of publications with 35 articles and Smith A.H. was the most cited author with the number of citations reaching 6987 times overall and 225 times per article. The most frequent keywords excluding the search subject were “arsenic”, “chlorination”, “trihalomethane”, and “disease agents”.

**Conclusion:**

This study is the first systematic bibliometric study of the literature publications on drinking water pollution and bladder cancer. It offers an overall and intuitive understanding of this topic in the past few years, and points out a clear direction research hotspots and reveals the trends for further in-depth study in future.

## Introduction

Bladder cancer is a common urological malignancy in females and the sixth most common type of urological malignancy in males; approximately 573,000 new cases and 213,000 deaths were reported in 2020 globally (1). Diverging incidence trends of bladder cancer have been observed; there were stabilizing or declining rates in males, but some increasing trends were observed for females (2, 3). Clinical studies have shown that the 5-year survival rate is approximately 90% although several patients with a first diagnosis of bladder cancer are treated with surgical resection combined with regular postoperative bladder infusion of chemotherapy drugs and other modern advanced treatments. However, most patients experience tumor recurrence within a few years after surgery, and up to 20% of patients with bladder tumor recurrence have lymph node metastasis and distant multiple metastases (4). The 5-year survival rate of patients with muscle-invasive bladder cancer is less than 50%, and muscle-invasive bladder cancer is more likely to metastasize (5). However, the etiology and mechanism of bladder cancer occurrence, recurrence, and metastasis are still unclear, which further impacts the incidence, prognosis, and mortality rates.

Studies have shown that the risk factors for bladder cancer include age, race, obesity, family history, and environmental risk factors, among others (6). Environmental risk factors are important factors leading to increased risks of bladder cancer and have attracted the interest of researchers (7, 8). For example, smoking increases the risk of bladder cancer and significantly increases the risk of bladder cancer recurrence and mortality in patients with non-invasive bladder cancer (2, 9). Occupational carcinogens (including exposure to diesel exhaust, polycyclic aromatic hydrocarbons, certain pesticides, and herbicides) (10) and previous exposure to chemotherapeutic agents (11) are risk factors for the development of bladder transitional cell carcinoma.

The bladder is the main excretion organ for urine, and a large number of toxic substances accumulate in it. Therefore, drinking water pollution, another potential risk factor for bladder cancer, has also attracted the interest of researchers in recent years (12, 13). For example, the long-term consumption of chlorinated drinking water containing complex mixtures leads to a significant increase in the incidence of bladder cancer as a by-product of chlorinated bromination in water (14). According to the U.S. Environmental Protection Agency, the highest concentration of arsenic in drinking water is 10 μg/L, which increases the risk of bladder cancer (15). Therefore, lifestyle interventions may be useful for the prevention of bladder cancer recurrence. However, systematic analyses on the role of drinking water pollution and bladder cancer in preventing bladder cancer recurrence are limited.

Bibliometrics is a scientific method that analyzes the distribution, quantitative relationship, and change rules of previous documents based on the literature system and bibliometric characteristics by applying various quantitative methods, such as mathematics and statistics (16). Bibliometric analysis can record the breadth and depth of the current status of scientific literature research and predict future research trends. Given its robustness, clarity, and comprehensiveness, it plays an important role in scientific bibliometrics. However, to the best of our knowledge, there have been no published bibliometric studies on drinking water and bladder cancer.

In this study, we conducted a systematic review using the method of bibliometric analysis to establish a visual knowledge map by searching the literatures related to drinking water and bladder cancer in the Scopus database, and summarize and analyze the literature publication, research topics, research hotspots and future development trends in this field, so as to provide a reliable reference of drinking water pollution and bladder cancer.

## Materials and methods

### Literature search and data acquisition

We searched the world’s largest Scopus database through the Wuhan University Library on January 7th, 2023, and the search strategy was set as the following: (1) search time: scheduled until January 7th 2023; (2) search terms: limited to “bladder cancer” and “ drinking water “; (3) search scope: “TITLE-ABS-KEY”; (4) literature types: select all, including regular and review articles, and other forms. With no limitation in publication language, all other settings were as the default value, and duplicate or invalid documents were removed. Thus, we obtained 687 published articles with a comprehensive coverage of all the available literature, studies pertaining to fields other than medicine, studies on non-human subjects, and those without abstracts were also included. We download all the articles information including but not limited to Author, Title, Cited by, Affiliations in RIS and CSV file formats for further analyses. In VOSviewer, we select the minimum number of documents of the nodes according to the needs of data visualization and set other documents as the default value.

### Software and version

We used text-mining R packets such as “NLP”, “tm”, “wordcloud2”, “ggplot2” to cluster title keyword and draw clustering diagrams. The version of R software is 4.1.0. The VOSviewer 1.6.18 software was used to visualize network of co-author, co-occurrence and co-citation. In addition, several tables in the study were generated in the Microsoft Excel program of Microsoft Corporation.

### Statistical analysis and visualization

**Figure 1:**
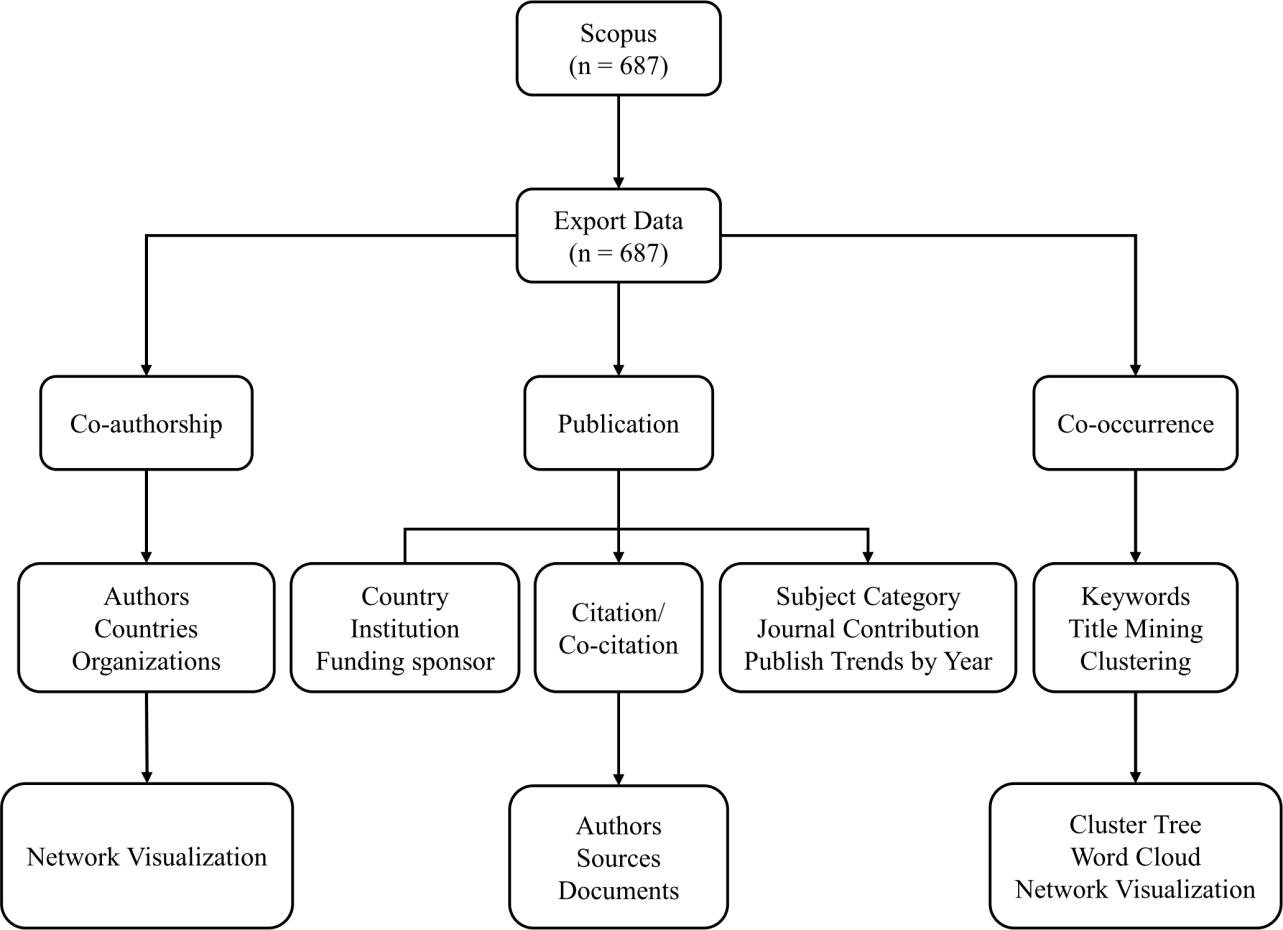
Workflow of the bibliometrics analysis.

In this bibliometrics analysis, we created network visualization using VOSviewer version 1.6.18 (released on January 24, 2022, Centre for Science and Technology Studies, Leiden University, Leiden, the Netherlands) with nodes representing documents, affiliations, authors, or keywords/author-keywords and can be connected by co-authorship, citation, co-citation, and co-occurrence analysis. The size of a node was determined by the weight of the element, such as the number of publications, citations, or the frequency of occurrences. And cluster of the same color indicated the same category, which was a set of items in the network with similar properties. The thickness of the links and the total link strength (TLS) were used to quantitatively assess the links. Similar analyses and visualizations were performed to create network map of highly cited documents and the journals, keywords, authors with the publications. The workflow of this bibliometrics analysis is shown in **Figure 1**.

## Results

### Bibliometric analysis of basic information on drinking water pollution and bladder cancer

**Figure 2:**
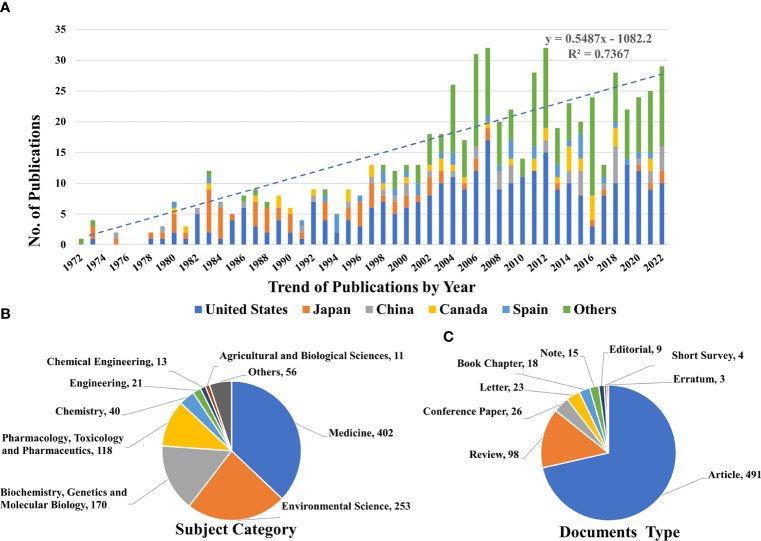
Basic Information of publishing trends and statistical results. **(A)** is the line graph and linear fitting curve showing the number of published documents each year. **(B)** shows the different subject categories of the published documents. **(C)** shows the documents types of the published documents.

We retrieved 687 documents related to drinking water and bladder cancer. The number of published documents has been increasing, but the rate has reduced in recent years. Based on the number of published articles, the top five countries were the United States, Japan, China, Canada, and Spain. Among them, the United States published approximately four times the number of articles published by second-ranked Japan, reaching 345 articles (**Figure 2A**). The number of published research articles in China and other countries has increased significantly within the past 20 years. It also shows that more countries have begun to pay attention to drinking water safety. Among the top 10 institutions with the most publications, seven were in the United States (**Supplementary Table 1**). Moreover, the top five funding sponsors were all in the United States (**Supplementary Table 2**). As observed, the literature dominantly belonged to the categories of medicine (n = 402) and environmental sciences (n = 253), which accounts for more than 60% of the documents that had duplicate disciplines. Reports in the fields of biochemistry, genetics, molecular biology, pharmacology, toxicology, pharmacy, chemistry, and other disciplines were also found (**Figure 2B**). Researchers are looking for causal relationships and progress for several aspects of drinking water pollution and bladder cancer. Most of the studies were research articles (n=491, 71.47%). There were also 98 review papers, 26 conference papers, and 23 letter papers; most of these were research-based statistical analysis documents (**Figure 2C**). With the development of the economy and society, the relationship between drinking water and bladder cancer has attracted increasing attention from scholars globally.

### Distribution analysis of journals, authors, and co-authorship in the fields

**Figure 3:**
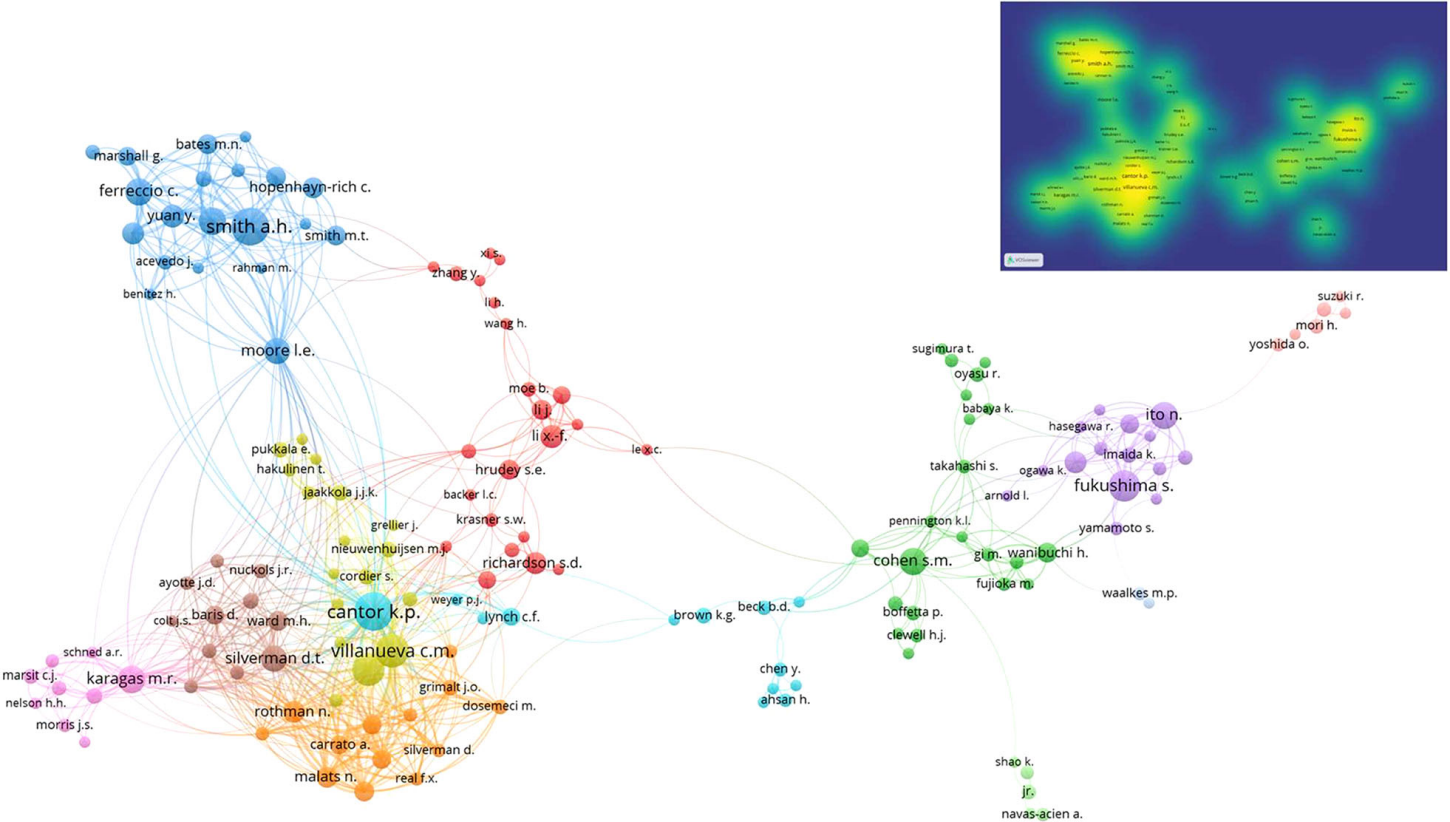
The network of co-authorship. In the network graph, there are ten colors representing different clusters, and the size of points represents the number. Some of the 197 items in the network are not connected to each other, so we replaced this 197 items network with 156 items that included the largest number authors. The upper left is the density map by publications.

To further investigate the data on journal publications and bibliographic citations, we performed a more specific bibliometric analysis and visualization of the source journals, authors, and co-authorship. As shown in **Table 1**, 152 journals were assessed, and 13 had published at least 10 papers. The journal with the most publications was *Environmental Health Perspectives*, with 46 published within half a century, which was 19 more than the number of publications of the second journal, the *American Journal of Epidemiology*. *Environmental Health Perspectives* was also the only journal with more than 15 publications. Eleven of the 13 journals that had published at least 10 papers belonged to the Q1 journal category according to the latest Thomson Web of Knowledge Journal Citation Reports Ranking, and their impact factors were within the range of 4–13. Six of these journals had impact factors exceeding 10. At the same time, 33 journals had published no less than five articles. Regarding the bibliometrics analysis of the author, **Table 2** shows the data of 10 authors with the most published articles in this field. Professor Cantor K.P. from the National Cancer Institute published 35 articles in this field, which was the highest number of publications. The most cited author was Smith A.H., with the number of citations reaching 6987 times overall and 225 times per article. Co-authorship is an important part of scientific research, from which we can identify clusters of authors and their contributions to publications in this field. **Figure 3** shows collaborative associations among 197 authors filtered from 2336 authors in total, with the minimum number of documents per author being 3. Focusing on the authors with the most published literature, 10 author groups were formed, among which the Cantor K.P. and Vilanueva C.M. cluster groups were the most closely related.

**Table 1:** Journals published no less than 10 items.

Sort	Journals	No. of documants	Impact Factor*	JCR*
1	**Environmental Health Perspectives**	46	11.035	Q1
2	**American Journal Of Epidemiology**	19	5.363	Q2
3	Toxicology And Applied Pharmacology	15	11.357	Q1
4	**Environmental Science And Technology**	14	4.46	Q2
5	Science Of The Total Environment	14	10.753	Q1
6	Journal Of The National Cancer Institute	13	11.816	Q1
7	Journal Of Urology	13	7.6	Q1
8	Cancer Research	12	13.312	Q1
9	International Journal Of Environmental Research And Public Health	12	4.614	Q1
10	Environmental Research	11	8.431	Q1
11	Epidemiology	11	4.86	Q1
12	**Environment International**	10	13.352	Q1
13	Toxicology	10	4.571	Q1

*Journal impact factor based on Thomson Web of Knowledge Journal Citation Reports Ranking(2022).

**Table 2:** Statistics of the 10 authors with the most published articles.

Author	No. of publications	Citations in 669-study	Publications in Web of Science	Sum of Times Cited*	Cited by articles in total*	H-index*
Cantor K.P.	35	3063	214	10245	7297	57
Smith A.H.	31	6987	212	15655	9872	69
Villanueva C.M.	25	2247	84	3883	2555	34
Fukushima S.	22	868	200	2935	2282	28
Kogevinas M.	21	2116	670	31007	23536	93
Cohen S.M.	17	979	182	4030	2724	38
Ferreccio C.	17	1436	236	18993	15799	58
Ito N.	17	715	17	95	94	5
Karagas M.R.	17	984	504	18513	13870	74
Steinmaus C.	17	1828	124	6342	4627	45

*Information from Author Profile webpage of Web of Science.

### Literatures citation and co-citation analysis

**Figure 4:**
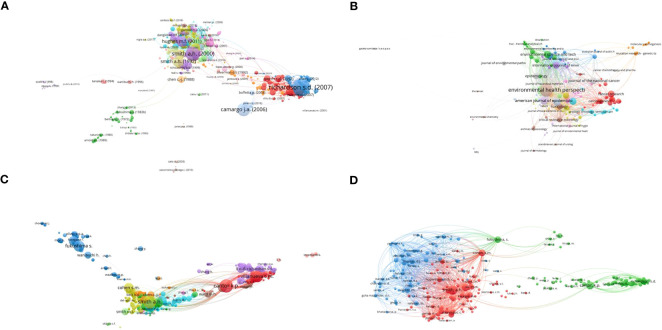
Network of citation of documents, journals and authors by VOSviewer. **(A-C)** represent the citations of documents, journals, and authors, respectively. **(D)** is the co-citation network of the cited authors. Document network shows the citation relationship between 420 documents in the name of authors. 170 journals appear in **(B)** D graph is a subset of C graph.

Citation is the most important indicator for evaluating the influence of a journal or article, and we analyzed and visualized the citations of journals and literature to fully understand the research situation in the field of drinking water pollution and bladder cancer. As shown in **Table 3**, we collected the basic information of the 10 most-cited articles and found that the most-cited document was cited 2330 times. Three of the top 10 articles came from the Smith A.H. team, which also confirmed the result that the Smith A.H. team received the highest number of citations in the study (**Table 2**). There were also four journals with publication number rankings, as shown in **Table 3**. Proceeding with the study citations and co-citations, the citations were presented from the perspectives of literature, journals, and authors. The number of documents shown in **Figure 4A** is 433 instead of all 687 articles, with the conditions that the minimum number of citations of a document is 5 and the largest set of connected items is 433. The inclusion criteria for the 289 journals are that the minimum number of documents of a source is 1 and the minimum number of citations of a source is 10. Finally, 189 journals met the thresholds (**Figure 4B**). The filter rules for 2320 authors were also similar; the minimum number of documents and citations of an author were 1 and 50, respectively, and 843 authors were selected from the network (**Figure 4C**). Overall, only 223 authors out of 41618 had been cited more than 50 times. **Figure 4D** shows that the aggregation effect was significant.

**Table 3:** Basic information of the top 10 documents in total citations.

Authors	Title	Year	Source Journal	Impact Factor*	Cited by	Affiliations
Richardson S.D. et al.	Occurrence, genotoxicity, and carcinogenicity of regulated and emerging disinfection by-products in drinking water: A review and roadmap for research	2007	Mutation Research - Reviews in Mutation Research	3.151	2330	National Exposure Research Laboratory, US Environmental Protection Agency, Athens, GA 30605, USA.
Smith A.H. et al.	Contamination of drinking-water by arsenic in Bangladesh: A public health emergency	2000	Bulletin of the World Health Organization	13.831	1557	Department of Epidemiology, School of Public Health, University of California, Berkeley, Berkeley, CA 94720-7360, United States.
Camargo J.A. et al.	Ecological and toxicological effects of inorganic nitrogen pollution in aquatic ecosystems: A global assessment	2006	**Environment International**	13.352	1367	Departamento de Ecología, Edificio de Ciencias, Universidad de Alcalá, 28871 Alcala de Henares, Madrid, Spain.
Smith A.H. et al.	Cancer risks from arsenic in drinking water	1992	**Environmental Health Perspectives**	11.035	947	Biomedical/Envtl. Health Sci. Dept., 314 Warren Hall, University of California, Berkeley, CA 94720, United States.
Hughes M.F. et al.	Arsenic exposure and toxicology: A historical perspective	2011	Toxicological Sciences	4.109	836	Office of Research and Development, National Health and Environmental Effects Research Laboratory, U.S. Environmental Protection Agency, Research Triangle Park, NC 27711, United States.
Chen C.-J. et al.	Cancer potential in liver, lung, bladder and kidney due to ingested inorganic arsenic in drinking water	1992	British Journal of Cancer	9.075	675	Institute of Public Health, National Taiwan University College of Medicine, Taipei, 10018, Taiwan.
Kitchin K.T.	Recent advances in arsenic carcinogenesis: Modes of action, animal model systems, and methylated arsenic metabolites	2001	**Toxicology and Applied Pharmacology**	4.46	672	Environmental Carcinogenesis Division, National Health and Environmental Effects Research Laboratory, U.S. Environmental Protection Agency, Research Triangle Park, NC 27711, United States.
Smith A.H. et al.	Marked increase in bladder and lung cancer mortality in a region of northern chile due to arsenic in drinking water	1998	**American Journal of Epidemiology**	5.363	652	School of Public Health, University of California, Berkeley, CA, United States.
Costa M.	Toxicity and carcinogenicity of Cr(VI) in animal models and humans	1997	Critical Reviews in Toxicology	6.184	494	Nelson Inst. of Environ. Medicine, Kaplan Cancer Center, 550 First Avenue, New York, NY 10016, United States.
Mohammed Abdul K.S.	Arsenic and human health effects: A review	2015	Environmental Toxicology and Pharmacology	5.785	455	Department of Zoology, Faculty of Science, University of Ruhuna, Matara, 81000, Sri Lanka

*Journal impact factor based on Thomson Web of Knowledge Journal Citation Reports Ranking (2022).

### Distribution analysis of keywords and network analysis of co-occurrence of the documents

**Figure 5:**
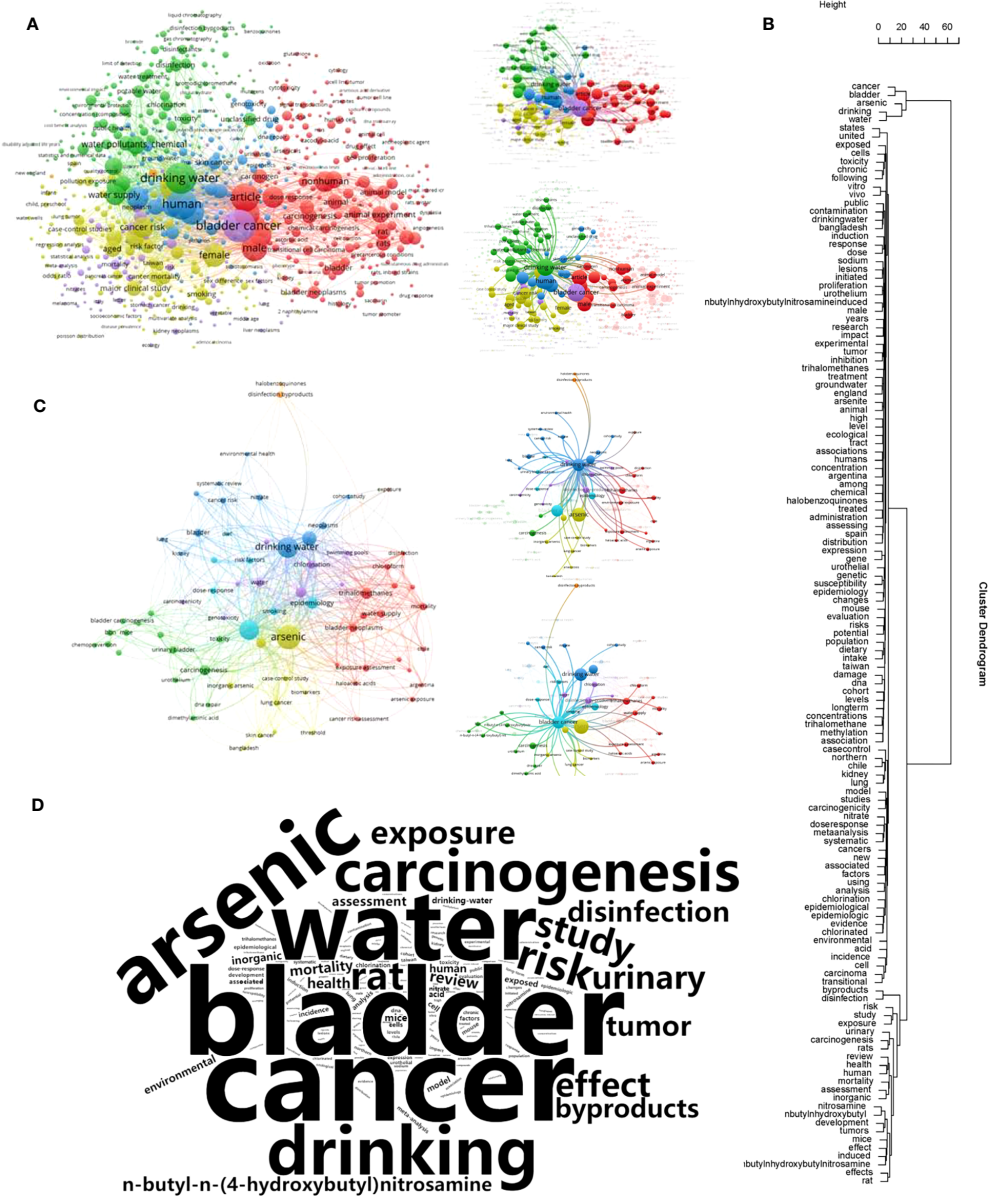
Keywords, author keywords and title test mining. **(A)** is the network of all keywords network and A right are two cluster based on ‘bladder cancer’ and ‘drinking water’. **(B)** shows the network of author keywords, and the B right are two diagrams displaying a more accurate clustering relationships based on ‘drinking water’ and ‘bladder cancer’. **(C)** is the cluster tree of all 669 titles by title test mining. **(D)** is the word cloud of the title keywords.

We included two types of keywords, all keywords and author keywords, for the bibliometric analysis. The former included the high-frequency keywords filtered from the title and abstract, and the author keywords were those provided by the author, usually including 5–8 words. We performed clustering and visualization based on the frequency of the co-occurrence of the keywords. For future analysis, the title text mining clustering tree and word cloud were generated by R software through the text mining package “tm.” The all-keyword analyses included 5433 keywords, with the filtering condition that the minimum number of occurrences of a keyword was 5. Finally, 734 keywords were selected. The highest-ranked contaminant was arsenic, which appeared 365 times, which is consistent with the reality of water pollution. The clustering results also showed that several toxicants associated with carcinogenicity in water pollution, such as arsenic, chlorination, and trihalomethane, and disease agents appeared in the bladder cancer-related clustering (**Figure 5A**). Of the total of 1167 author keywords, only 74 appeared more than five times, but the network clustering results showed important information. Based on the clustering results, drinking water pollution was highly associated with skin cancer, in addition to bladder cancer. Cluster analysis of the title keywords revealed more concentrated terms with causal associations (**Figure 5B**). **Figure 5C** shows the type of toxic substances involved in drinking water pollution and the highly related organ damage, such as the bladder, liver, lung, and kidney. Damage pathway analyses were also performed, which revealed methylation and genetic mutations. To show the frequency of title keywords more intuitively, we used the title text-mining results to generate a word cloud map (**Figure 5D**). We found the high-frequency entries and topics that had been studied in the field in this cloud map. Larger fonts denoted higher frequencies and importance. This visualization can help researchers quickly obtain the distribution of popular research results in this field.

## Discussion

In this bibliometric analysis, we systematically analyzed the essential bibliometric parameters, such as the number and type of publication articles, institutions, funding sponsor distribution, and journals of study associated with the relationship between drinking water and bladder cancer, and identified the journals with the highest number of published manuscripts and/or citations. We created the co-authorship network to identify the collaborations among authors, countries, and organizations. We drew a citation/co-citation point-like relationship diagram to describe the linkage of authors, documents, and journals. Citation/co-citation precedes the interconnection between studies in this field. To identify the hot topics related to the relationship between drinking water pollution and bladder cancer, we generated the term cloud. In short, through the above analysis, we gained a comprehensive and in-depth understanding of research in this field and assessed its development and current situation, which will be useful in further promoting the depth and breadth of future research (17).

The number of published articles in a particular field is an important indicator of the development of scientific research. This reflects the change in the knowledge of scholars on the subject, as well as the latest research progress in this field (18). We collected 687 articles related to drinking water and bladder cancer published between 1972 and 2023 from the largest Scopus literature database, from which the number of research studies on drinking water and bladder cancer is on the rise, and the subject areas and research hotspots are constantly expanding. For the first report on drinking water and bladder cancer from 1972 to 2001, there were no more than 13 publications in the field annually. However, since 2002, the number of documents published in the field has grown rapidly; there were 32 publications in 2007. In addition, we established a simple growth trend model (y=0.515x-1015.2, R^2 = ^0.6939) that could predict the publication of articles, and it showed that as many as 28 articles on drinking water pollution causing bladder cancer will be published in 2023. Overall, the number of published documents has increased, although the growth rate has decreased in recent years. Several countries have recently appeared on the list of posts and their publications are increasing every year. This also shows that an increasing number of countries are focusing on the safety of drinking water. This may be due to the fact that researchers in countries that initially studied drinking water pollution are still limited to the identified pollutants, and no breakthrough has been made in other possible drinking water pollutants, pathogenic mechanisms, and improvement of exposure assessment and accurate characterization of individual factors, while developing countries pay more and more attention to the relationship between drinking water pollution and diseases.

The level of national emphasis and scientific research on sanitation and health are often closely related to local economic and social development and public health status (19, 20). The present study demonstrated that the United States, Japan, Canada, and Spain, as traditionally developed countries, have been the main countries involved in the study of the relationship between drinking water pollution and bladder cancer. Surprisingly, China (21) and other countries (22, 23) have also paid more attention to it within the past 20 years, accounting for more than half of the articles published every year. For example, the Chinese government has recently revised the Environmental Quality Standards for Surface Water (EQSSW) (GB3838-2002) to address the challenges of environmental protection by refining water function zoning, establishing priority pollutants, and improving the protection of drinking water sources (24). Furthermore, the United States is still at the forefront based on the perspective of research institutions and fund sponsors, which means that American research institutions have a strong scientific output in this field. This may be related to the high incidence of bladder cancer in North America according to the global cancer statistics (1, 25), and they are more concerned and eager to determine the relationship between water pollution and bladder cancer, as well as prevent it.

From the perspective of research by discipline category, current research is mainly concentrated in the field of medicine and environmental science, accounting for more than 60% of the subject double-counting literature. This is consistent with our prediction of the association between drinking water pollution and bladder cancer development. Additionally, there are research reports in the fields of biochemistry, genetics, molecular biology, pharmacology, toxicology, pharmacy, chemistry, and other disciplines. This suggests that researchers around the world are searching for causal relationships and progress on several aspects of this association (12, 13).

From the perspective of research output, the number of studies on drinking water and bladder cancer is on the rise, and the subject areas and research hotspots are constantly expanding. Among the high-yield journals, *Environmental Health Perspectives* published most of the articles. This indicates that research in the field of drinking water pollution and bladder cancer has been widely recognized, and corresponding preventive measures have been focused on. However, in general, the number of publications in high-quality core journals remains small. The authors with the high number of publications were Professor Cantor K.P. from the National Cancer Institute and Professor Smith A.H. from the University of California, who have long been engaged in epidemiological (26) and risk factor (27) research on the relationship between pollutants in drinking water and cancer. This field has several groups of authors with high yields and some high-yield research teams have a certain degree of cooperation. However, in-depth cooperation is lacking.

To better understand the current situation and research development for drinking water pollution and bladder cancer, a visual analysis of drinking water pollution and bladder cancer was performed and the research hotspots in this field were determined. The clustering results also showed that several toxicants associated with carcinogenicity in water pollution, such as arsenic (28), chlorination (29), trihalomethane (30), and disease agents (31), appeared in the bladder cancer-related clustering. From the clustering results, drinking water pollution was highly associated with skin cancer (32), in addition to bladder cancer. Cluster analysis of the title keywords showed more concentrated terms with causal associations; drinking water pollution and highly related damage to organs, such as the bladder, liver, lung, and kidney. There are also damage pathway analyses, with methylation and genetic mutations appearing, which provide a guide for future research.

Future ahead, with continuous research in this field, the studies on drinking water pollution and bladder cancer will steadily increase. Several experimental, clinical, and evidence-based studies have elucidated the association between drinking water pollution and bladder cancer from different perspectives. However, there remains a regional imbalance in available information for the study of drinking water pollution and bladder cancer. Additionally, there is an insufficient collaboration between research groups. Therefore, we suggest that researchers can combine data mining (21), artificial intelligence (33), and other computer technologies or meta-analysis (34) to conduct interdisciplinary research, which will further improve the research methods and broaden ideas, provide a scientific basis for epidemiological and clinical oncology research on bladder cancer decision-making, and provide a new direction for the discussion of drinking water pollution and bladder cancer prevention and treatment.

Our study has some limitations. First, we only used the Scopus database to search publications related to drinking water and bladder cancer. Although Scopus database, with tremendous information on authors, countries, journals, and citations distribution, is the most widely used tool for bibliometric analysis, some influential literatures from other databases (namely WoSCC, SCI, EI and SSCI) may have been excluded, resulting in a small amount of bias. Second, the literature in Scopus database is predominantly published in English language. Therefore, this study may lack high-impact articles written in non-English languages. Third, this study only conducted a preliminary bibliometric analysis of drinking water and bladder cancer; however, the research methodology of existing literatures and carcinogenic mechanism of pollutants in drinking water were not thoroughly studied. Hence, we will conduct a further in-depth analysis. Despite the above shortcomings, we believe that our findings can provide valuable suggestions for future research development of this field.

## Conclusion

Bibliometric analysis of drinking water and bladder cancer has a significant guiding role in the research of water resources, water environment, and incidence of bladder cancer. We drew landscapes of publication year, affiliation, citation/co-citation, source journal, author/co-authorship, keywords, and title text to find key research hotspots. The most popular research direction is disinfection by-products causing bladder cancer, which confirms the importance of the bibliometric analysis of drinking water pollution in epidemiological and clinical oncology research.

## Data availability statement

The original contributions presented in the study are included in the article/**Supplementary Material**. Further inquiries can be directed to the corresponding authors.

## Author contributions

JW and SL: study conception and design. YZ, ML, JJW and SX: data acquisition. KXH, FYH and BCW: analysis and data interpretation. YZ and JW: drafting of the manuscript. JW, SL, MDZ and CHY: review and revise the manuscript. All authors contributed to the article and approved the submitted version.
